# Expression Patterns and Prognostic Values of ORMDL1 in Different Cancers

**DOI:** 10.1155/2020/5178397

**Published:** 2020-10-21

**Authors:** Tengjiao Zhu, Yingtong Chen, Shuyuan Min, Fang Li, Yun Tian

**Affiliations:** Peking University Third Hospital, 49 Huayuan North Road, Beijing 100191, China

## Abstract

The mammalian orosomucoid-like gene family (*ORMDL*), containing *ORMDL1*, *ORMDL2*, and *ORMDL3*, is the important regulator of sphingolipid metabolism, which is relevant to cell growth, proliferation, migration, and invasion. Since the role of *ORMDL1* in cancers remained unclear, the main purpose of our study was to explore the expression patterns and prognostic values of *ORMDL1* in different tumors, especially in cholangiocarcinoma (CHOL), lymphoid neoplasm diffuse large B cell lymphoma (DLBCL), acute myeloid leukemia (LAML), and thymoma (THYM). Bioinformatics tools including GEPIA, CCLE, LinkedOmics, cBioPortal, and TIMER databases were used. As a result, the expression levels of *ORMDL1* in tumor tissues and normal tissues varied in different cancers, especially significantly upregulated in CHOL, DLBCL, LAML, and THYM. Moreover, *ORMDL1* mRNA was also highly expressed in cell lines of DLBCL and LAML. Further studies showed that *ORMDL1* overexpression was associated with poor prognosis in DLBCL, but not significant in CHOL, LAML, and THYM. Consistently, there were genetic alterations of *ORMDL1* in DLBCL, and patients with genetic alterations indicated worse survival. Coexpressed genes and related biological events with *ORMDL1* in DLBCL were found via LinkedOmics, Gene Ontology (GO), and Kyoto Encyclopedia of Genes and Genomes (KEGG) analysis. The relationship between *ORMDL1* and cancer immune cells was investigated, and *ORMDL1* expression was positively correlated with infiltrating levels of B cells. In conclusion, *ORMDL*1 is suggested to be a tumorigenic factor and considered as the potential therapeutic target and prognostic biomarker in DLBCL.

## 1. Introduction

The mammalian orosomucoid-like gene family (*ORMDL*), including *ORMDL1*, *ORMDL2*, and *ORMDL3*, encodes transmembrane proteins localized to the endoplasmic reticulum [1–5]. *ORMDLs* are primarily involved in negative feedback regulation of sphingolipid metabolism, ceramide synthesis, and unfolded protein response [3–10]. The full-length human *ORMDL1* cDNA was originally obtained after screening a human retinal cDNA library and confirmed to be located at chromosome 2q32 [1]. During the process of sphingolipid biosynthesis, serine palmitoyltransferase (SPT) catalyzed the critical rate-limiting step. Inhibition of *ORMDL1* led to enhanced SPT activity and increased sphingolipid levels [6]. In addition, the expression levels of *ORMDL1* were demonstrated to be significantly correlated with familial Alzheimer's disease-related presenilin (PS) mutations, manifesting as elevated *ORMDL1* and *ORMDL2* levels due to PS deficiency. Silencing of *ORMDLs* suppressed nicastrin maturation and *γ*-secretase function [2]. For *ORMDL3*, it was closely associated with asthma risk in childhood [11, 12] and participated in cellular stress response [13], lymphocyte activation [14], and eosinophil trafficking [15].

As an important component of the cell membrane, sphingolipids can regulate cell growth, proliferation, migration, invasion, and metastasis through cancer signaling pathways, in addition to exerting barrier function and maintaining membrane fluidity [16–18]. Since *ORMDL1* was a regulator of sphingolipid levels in cells, we hypothesized that *ORMDL1* might play a role in the pathogenesis and progression of tumors. Thus, our study shed light on the specific role of *ORMDL1* in different tumors via bioinformatics analysis, particularly in cholangiocarcinoma (CHOL), lymphoid neoplasm diffuse large B cell lymphoma (DLBCL), acute myeloid leukemia (LAML), and thymoma (THYM). We emphatically investigated the expression level of *ORMDL1* in different types of cancers, the effect of *ORMDL1* expression on patient prognosis, and the genetic alterations and the potential interaction of *ORMDL1* with related genes especially in DLBCL.

## 2. Methods

### 2.1. GEPIA Dataset Analysis

Gene Expression Profiling Interactive Analysis (GEPIA, http://gepia.cancer-pku.cn/index.html) is an online database. As an interactive web, GEPIA provides 9,736 tumors and 8,587 normal samples from The Cancer Genome Atlas (TCGA) and the Genotype-Tissue Expression (GTEx) projects for analyzing the RNA sequencing expression data [19]. GEPIA was used to analyze the differential expression of *ORMDL1* between normal tissues and tumor tissues in 33 different types of cancer. We compared the expression patterns of *ORMDL1* in four cancer types, including CHOL, DLBCL, LAML, and THYM. Moreover, GEPIA also provided the function for prognostic curve analysis and pathological stage evaluation.

### 2.2. CCLE Dataset Analysis

The Cancer Cell Line Encyclopedia (CCLE, http://www.broadinstitute.org/ccle/home) project is a collaboration between the Broad Institute and the Novartis Institutes for Biomedical Research and its Genomics Institute of the Novartis Research Foundation. It can be applied to conduct a detailed genetic and pharmacologic characterization of a large panel of human cancer models, develop integrated computational analyses that link distinct pharmacologic vulnerabilities to genomic patterns, and translate cell line integrative genomics into cancer patient stratification. CCLE provides public access to genomic data, analysis, and visualization for about 1,000 cell lines [20]. The expression of *ORMDL1* was verified by the CCLE dataset.

### 2.3. LinkedOmics Dataset Analysis

LinkedOmics (http://www.linkedomics.org/login.php) provides a newly developed platform for analyzing large-scale cancer omics data from TCGA and Clinical Proteomic Tumor Analysis Consortium (CPTAC) [21]. We used LinkedOmics to inquire into the prognostic values of *ORMDL1* expression in the four cancer types, including CHOL, DLBCL, LAML, and THYM. The survival differences were visualized by Kaplan–Meier plots. Furthermore, the correlation coefficient and coexpressed gene patterns were calculated according to the online instruction.

### 2.4. cBioPortal Analysis

The cBioPortal database (http://cbioportal.org) is an online database that converts complex cancer genomic data from TCGA into well-understood genetic, epigenetic, and proteomic data, including somatic mutations, altered copy number, mRNA and miRNA expression, DNA methylation, and protein abundance data. It can be used to explore genetic changes in tumor samples and compare the effects of these changes on patient survival [22]. In our study, 48 DLBCL samples (TCGA, Provisional) with pathological reports were selected for further analysis of *ORMDL1* genetic alterations using cBioPortal. The mutation plots were drawn to directly reflect all types of *ORMDL1* genetic alterations. Additionally, Kaplan–Meier survival curves were constructed to analyze the influence of *ORMDL1* genetic alterations on the DLBCL patient survival.

### 2.5. TIMER Analysis

Tumor Immune Estimation Resource (TIMER) is a comprehensive database for systematical analysis of the abundances of six immune infiltrates (B cells, CD4+ T cells, CD8+ T cells, neutrophils, macrophages, and dendritic cells) in diverse cancer types. The function of the gene module is to explore the correlation between gene expression and abundance of immune infiltrates [23]. In this study, the relationship between *ORMDL1* expression and the six immune cells was estimated by TIMER in DLBCL.

### 2.6. Statistical Analysis

The difference in *ORMDL1* expression between tumor tissues and normal tissues was compared with an independent *t*-test. *ORMDL*1 expression in different clinical stages was evaluated using one-way ANOVA. The relationship between *ORMDL1* expression and patient prognosis was detected using the Kaplan–Meier survival analysis and log-rank test. The correlation between *ORMDL1* and related genes was analyzed using the Pearson correlation test. *P* < 0.05 indicated statistical significance.

## 3. Results

### 3.1. Expression Levels of *ORMDL1* in Different Types of Human Cancers

**Figure 1:**
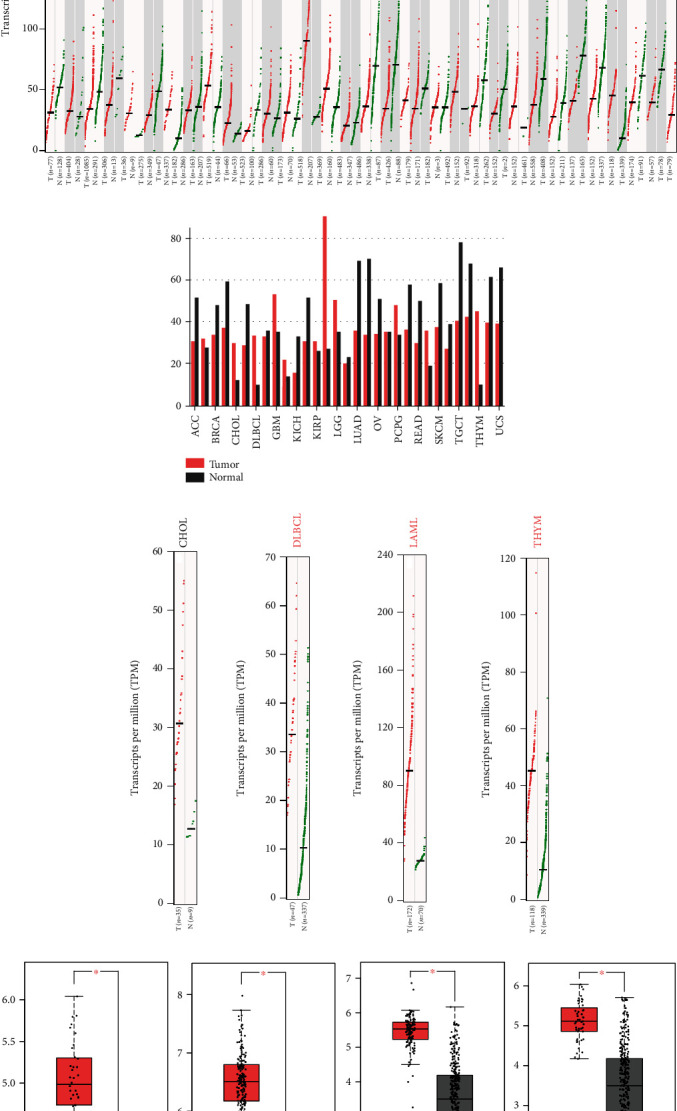
The expression levels of *ORMDL1* in CHOL, DLBCL, LAML, and THYM (GEPIA). (a, b) The expression levels of *ORMDL*1 in pan-cancer. (c, d) The expression levels of *ORMDL1* in CHOL, DLBCL, LAML, and THYM. ^∗^*P* < 0.05. CHOL: cholangiocarcinoma; DLBCL: lymphoid neoplasm diffuse large B cell lymphoma; LAML: acute myeloid leukemia; THYM: thymoma.

To determine differences of *ORMDL1* expression between tumor samples and normal samples, the *ORMDL1* mRNA levels of different tumor samples and normal samples were analyzed using GEPIA. The differential expression of *ORMDL1* in tumor samples and normal samples from all TCGA cancer types is listed in Figures 1(a) and 1(b). In particular, the results indicated that *ORMDL1* expression levels were significantly upregulated in CHOL, DLBCL, LAML, and THYM compared to their corresponding normal tissues (Figures 1(c) and 1(d)).

### 3.2. *ORMDL1* mRNA Expression in Different Kinds of Cancer Cell Lines

**Figure 2:**
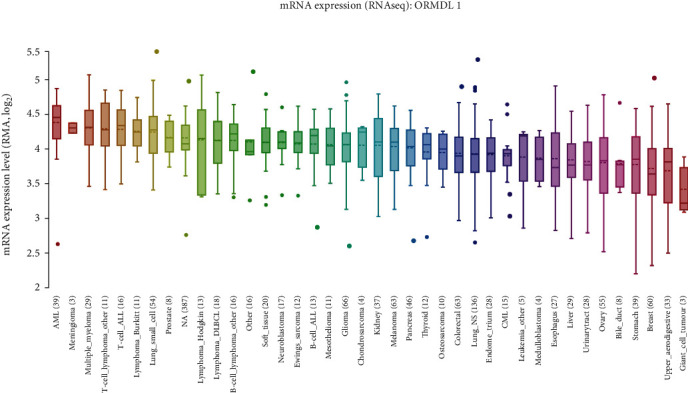
The expression of *ORMDL1* in cell lines of different cancer types (CCLE).

By collecting genetic information from CCLE, investigation of *ORMDL1* expression was extended to various cancer cell lines. As a result, *ORMDL1* mRNA was found to be highly expressed in cell lines of LAML and DLBCL, which ranked 1^st^ and 11^th^ among 40 kinds of cancers (Figure 2).

### 3.3. The Prognostic Influence of *ORMDL1* on CHOL, DLBCL, LAML, and THYM

**Figure 3:**
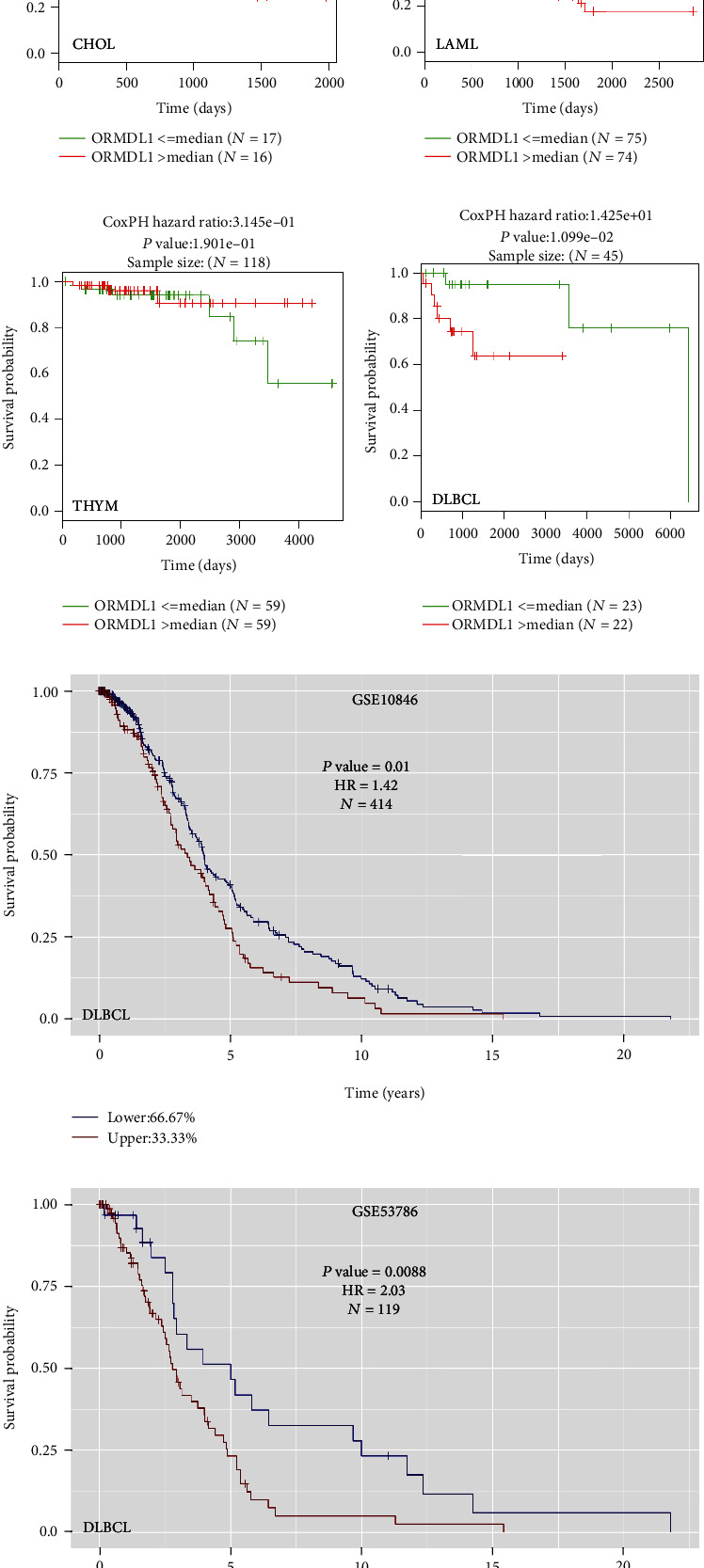
The prognostic value comparing the high and low expression of *ORMDL1* in CHOL, LAML, THYM, and DLBCL (LinkedOmics and shinyGEO). (a–d) Overall survival curves of CHOL, LAML, THYM, and DLBCL, analyzed by LinkedOmics. (e, f) Overall survival curves of DLBCL, analyzed by shinyGEO. CHOL: cholangiocarcinoma; LAML: acute myeloid leukemia; THYM: thymoma; DLBCL: lymphoid neoplasm diffuse large B cell lymphoma.

We further explored the influence of *ORMDL1* expression levels on the survival of patients in four cancer types, including CHOL, DLBCL, LAML, and THYM. The Kaplan–Meier curves and log-rank test analysis revealed that increased *ORMDL1* was associated with poor overall survival (OS) in DLBCL significantly, but not in CHOL, LAML, and THYM by LinkedOmics (Figures 3(a)–3(d)). Also, similar results were predicted in GSE10846 and GSE53786 using shinyGEO online tool, which could analyze patient survival from the GEO database, suggesting that DLBCL patients with higher *ORMDL1* levels tended to have lower OS (Figures 3(e) and 3(f)).

### 3.4. Genetic Alterations of *ORMDL1* in DLBCL

**Figure 4:**
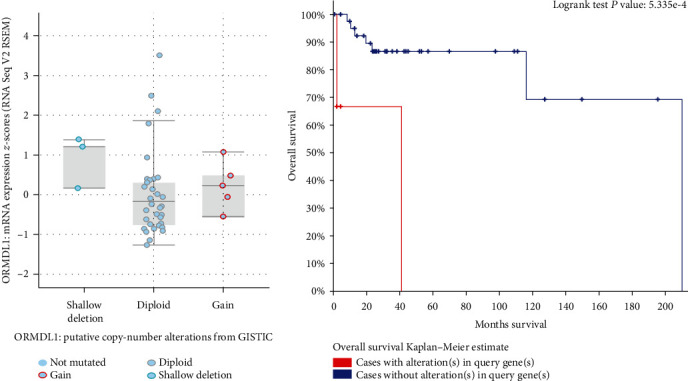
Genetic alterations of *ORMDL1* in DLBCL (cBioPortal). (a) *ORMDL1* mutation analysis in DLBCL. (b) Overall survival curves with or without *ORMDL1* alterations in DLBCL. DLBCL: lymphoid neoplasm diffuse large B cell lymphoma.

Since *ORMDL1* might play a role in DLBCL, genetic alterations of *ORMDL1* in DLBCL were determined using cBioPortal database analysis. *ORMDL1* mutations included gene gain and shallow deletion from 48 DLBCL patients (TCGA, Provisional) (Figure 4(a)). The relationship between *ORMDL1* genetic alterations and DLBCL patient survival was further evaluated. The Kaplan–Meier survival analysis showed that cases with genetic alterations were associated with worse prognosis (Figure 4(b)).

### 3.5. Coexpressed Genes and Functional Analysis of *ORMDL1* in DLBCL

**Figure 5:**
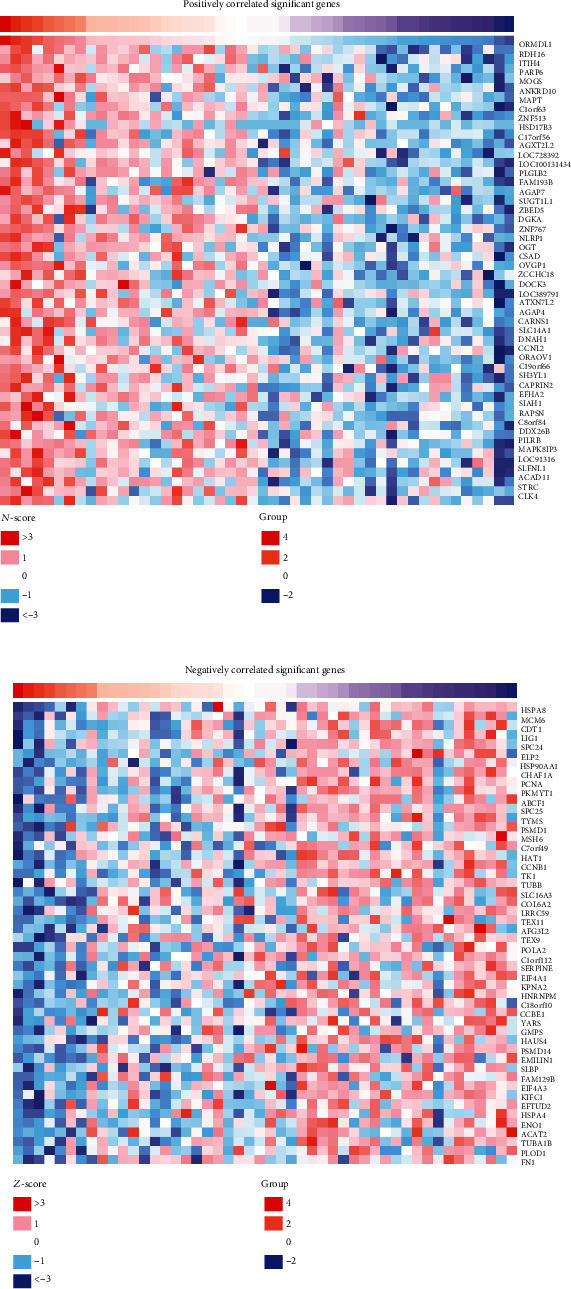
Coexpressed gene patterns of *ORMDL1* in DLBCL (LinkedOmics). DLBCL: lymphoid neoplasm diffuse large B cell lymphoma.

**Figure 6:**
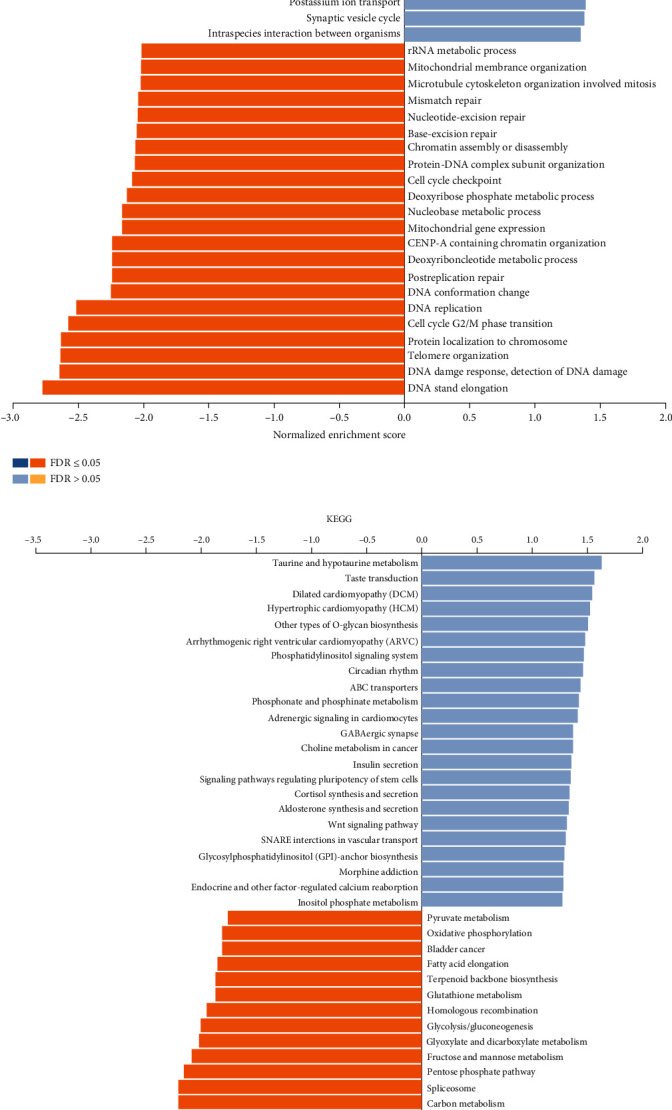
Functional analysis of *ORMDL1* in DLBCL. (a) Gene Ontology (GO) analysis indicated *ORMDL1* mainly participated in biological events like DNA damage response, nucleus localization, rRNA metabolic process, and cell cycle checkpoint. (b) Kyoto Encyclopedia of Genes and Genomes (KEGG) pathway analysis showed *ORMDL1* enriched in cell cycle, ABC transporters, oxidative phosphorylation, and DNA replication. DLBCL: lymphoid neoplasm diffuse large B cell lymphoma.

To figure out the potential interaction of *ORMDL1* with other genes in DLBCL, correlation analysis between *ORMDL1* and various genes and markers was performed via LinkedOmics. As shown in Figure 5, the top 50 significant genes positively and negatively correlated with *ORMDL1* were shown in the heat map. A detailed description of the coexpression genes is listed in Table 1. Furthermore, Gene Ontology (GO) analysis in biological process by GSEA indicated that *ORMDL1* coexpressed genes mainly participated in DNA damage response, nucleus localization, rRNA metabolic process, and cell cycle checkpoint (Figure 6(a)). Kyoto Encyclopedia of Genes and Genomes (KEGG) pathway analysis showed enrichment in cell cycle, ABC transporters, oxidative phosphorylation, and DNA replication (Figure 6(b)).

### 3.6. *ORMDL1* Is Correlated with Immune Infiltration Level in DLBCL

**Figure 7:**
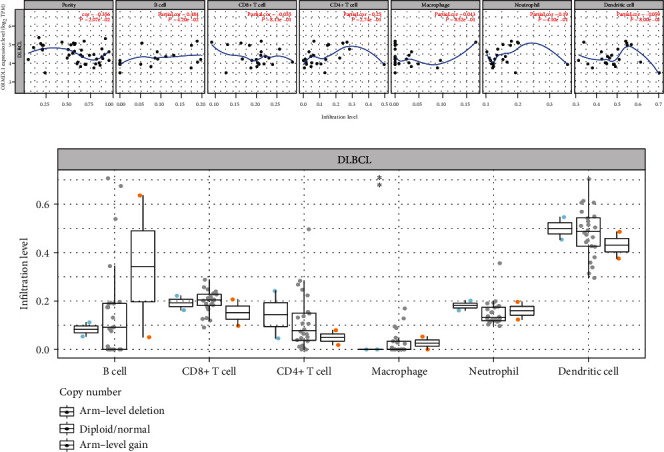
The relationship between *ORMDL1* expression and immune signatures. (a) The expression level of *ORMDL1* was significantly correlated with the infiltrating level of B cells in DLBCL. (b) The gene gain mutation of *ORMDL1* promoted the B cell infiltration in DLBCL. DLBCL: lymphoid neoplasm diffuse large B cell lymphoma.

To understand the relationship between *ORMDL1* expression and immune signatures, we analyzed the six immune marker genes in DLBCL, including B cells, CD8+ T cells, CD4+ T cells, macrophages, neutrophils, and dendritic cells. The results revealed that the expression level of *ORMDL1* was significantly correlated with the infiltrating level of B cells in DLBCL (Figure 7(a)). Moreover, the gene gain mutation of *ORMDL1* promoted the B cell infiltration in DLBCL (Figure 7(b)).

## 4. Discussion

Actually, the *ORMDL* gene family is a group of evolutionary conserved gene sequence found in *Drosophila*, yeast, and mammals. Among them, *Drosophila* only has a single-copy gene, yeast has two homologous genes of *Orm1* and *Orm2*, and mammalian cells contain three homologous genes with *ORMDL1*, *ORMDL2*, and *ORMDL3* [1]. The three human *ORMDL* isoforms are located on chromosomes 2q32, 12q13, and 17q21, respectively, with approximately 80% identical amino acid, proving that they may have some common biological functions [1, 12, 24]. The most important function of *ORMDLs* is to regulate sphingolipid biosynthesis and maintain ceramide homeostasis [3–10, 25–28], where SPT is the rate-limiting enzyme. In yeast, the *Orm*/SPT compound regulates sphingolipid expression levels by a negative feedback response. When the sphingolipid concentration is high, the *Orm* protein binds to SPT to inhibit SPT activity and reduce the further synthesis of sphingolipid. When the sphingolipid concentration is low, the N-terminal region phosphorylation of *Orm* protein causes its separation from SPT, which eliminates the repression of SPT and promotes sphingolipid biosynthesis [3, 4, 24, 25]. However, the regulation mechanism similar to that of yeast cannot be found in mammalian *ORMDLs* since human *ORMDL* proteins lack N-terminal phosphorylation sites [1, 4]. The study by Siow and Wattenberg demonstrated the feedback response of *ORMDL*-mediated sphingolipid synthesis [10]. The phenomenon that inhibition of SPT activity caused by permeable cells treated with C6 ceramide suggested *ORMDLs* might have a structural domain interacting with C6 ceramide, which was further involved in the regulation of *ORMDL*-dependent SPT activity. In addition, Wang et al. explored the relationship between *ORMDL1*, SPT, and sphingomyelin based on a free cholesterol- (FC-) loading microenvironment in human atherosclerotic macrophages [7, 9]. According to their research, the induction of endoplasmic reticulum (ER) stress and autophagy in FC-loaded macrophages led to *ORMDL1* shifting from ER to autophagosome, followed by the dissociation of SPT, which was originally bound to *ORMDL1*. Then, the activation of SPT resulted in increased sphingomyelin synthesis, excessive FC buffering, and reduced cytotoxicity.

Dysregulation of sphingolipid metabolism in cancers has been described in several studies [29–35]. Typical sphingolipid metabolites such as ceramide and sphingosine could be used as bioactive signaling molecules, suppressing cell growth and promoting apoptosis [31]. Phosphorylated metabolites such as sphingosine-1-phosphate (S1P) are related with survival, proliferation, and migration of cancer cells [36, 37]. The metabolism of bioactive sphingolipids in mammals is regulated by around 40 enzymes, which play key roles in cancer signaling pathways and therapeutic targets [29, 31, 32]. Consistently, sphingolipid enzymes and metabolites were abnormally expressed in a variety of cancers. For example, ceramide levels were upregulated in head and neck cancer and breast cancer [38, 39], while they were downregulated in ovarian cancer and colon cancer [40, 41]. Moreover, sphingosine was highly expressed in endometrial cancer [42]; S1P was overexpressed in glioblastoma [43], and SPT was lowly expressed in colon cancer [44]. Therefore, it was reconfirmed that metabolic disorders of sphingolipids interacted closely with tumorigenesis, tumor development, and chemoresistance of cancer patients.

As described above, *ORMDLs* act as critical factors in maintaining the balance of cellular sphingolipid levels. However, it remains unclear whether *ORMDLs* are involved in cancer networks associated with sphingolipid metabolism. In our study, we first preliminarily analyzed *ORMDL1* expression in tumor tissues and normal tissues using the GEPIA database and found that *ORMDL1* was expressed differently in diverse cancer tissues and adjacent tissues, especially highly expressed in CHOL, DLBCL, LAML, and THYM. Second, high expression of *ORMDL1* in cell lines of DLBCL and LAML was verified by the CCLE database, which was consistent with the results in the corresponding tumor samples. To further elucidate the prognostic effect of *ORMDL1* expression on CHOL, DLBCL, LAML, and THYM, the Kaplan–Meier survival curves were generated by GEPIA. It revealed that *ORMDL1* overexpression was significantly associated with poor survival of DLBCL, indicating *ORMDL1* might facilitate tumorigenesis and recurrence in DLBCL. The results of GSE10846 and GSE53786 further confirmed the results that high expression of *ORMDL1* indicated poor prognosis in DLBCL patients. In addition, the cBioPortal database was used as a powerful tool for discovering *ORMDL1* genetic alterations in DLBCL, since genetic alteration was considered as an important factor in cancer development [45, 46]. Expectedly, increased gene copies and slight gene deletion existed in DLBCL, which partly explained the higher expression of *ORMDL1* in DLBCL compared with normal samples. The concomitant result also showed cases with *ORMDL1* genetic alterations had worse prognosis. Finally, through LinkedOmics and Pearson correlation test, genes that positively and negatively interacted with *ORMDL1* were found and functional analysis in GO and KEGG pathways was further explored, which might be jointly involved in the *ORMDL1*-related cancer signaling pathways.

There were still some limitations to be solved. Firstly, differences of sample sizes among multidatabases might cause some bias. Secondly, this study only analyzed transcriptional levels of *ORMDL1* in cancers, without its posttranslational levels. Finally, molecular mechanism investigation should be carried out to further explore cancer pathways associated with *ORMDL1* and sphingolipid metabolism.

## 5. Conclusions

This was the initial study comprehensively analyzing the expression patterns and prognostic values of *ORMDL1* in different tumors. *ORMDL1* is promising to be the potential therapeutic target and prognostic marker in DLBCL.

## Figures and Tables

**Table 1:** Correlation analysis between *ORMDL1* and related genes in DLBCL by LinkedOmics.

Gene names	DLBCL	
	*Pearson*	*P* value
RBM6	3.736*e* − 01	8.902*e* − 03
ELMOD3	5.138*e* − 01	1.876*e* − 04
ANKRD13D	4.768*e* − 01	6.130*e* − 04
C1orf63	6.451*e* − 01	7.428*e* − 07
SFRS18	4.299*e* − 01	2.747*e* − 03
STX16	4.791*e* − 01	5.704*e* − 04
SUPT7L	3.958*e* − 01	5.358*e* − 03
DCUN1D2	3.992*e* − 01	4.943*e* − 03
POLA2	−4.732*e* − 01	6.819*e* − 04
SFRS17A	4.085*e* − 01	3.944*e* − 03
TMC8	4.460*e* − 01	1.486*e* − 03
SORBS1	4.469*e* − 01	1.452*e* − 03
PPWD1	4.299*e* − 01	2.294*e* − 03

## Data Availability

Data can be available upon request.
